# The importance of cell culture parameter standardization: an assessment of the robustness of the 2102Ep reference cell line

**DOI:** 10.1080/21655979.2020.1870074

**Published:** 2021-01-11

**Authors:** James Willard Tonderai Kusena, Maryam Shariatzadeh, Adam James Studd, Jenna Rebekah James, Robert James Thomas, Samantha Loiuse Wilson

**Affiliations:** a Centre for Biological Engineering, Wolfson School of Mechanical, Electrical and Manufacturing Engineering, Loughborough University, Loughborough, Leicestershire, UK; b Stem Cell Glycobiology Group, Division of Cancer & Stem Cells, School of Medicine, University of Nottingham, Queen’s Medical Centre, Nottingham, UK

**Keywords:** Cell Growth Rates, defined Cell Therapy Manufacturing, metabolites, reference Cell Lines, quality Control

## Abstract

Work undertaken using the embryonic carcinoma 2102Ep line, highlighted the requirement for robust, well-characterized and standardized protocols. A systematic approach utilizing ‘quick hit’ experiments demonstrated variability introduced into culture systems resulting from slight changes to culture conditions (route A). This formed the basis for longitudinal experiments investigating long-term effects of culture parameters including seeding density and feeding regime (route B).Results demonstrated that specific growth rates (SGR) of passage 59 (P59) cells seeded at 20,000 cells/cm^2^ and subjected to medium exchange after 48h prior to reseeding at 72h (route B2) on average was marginally higher than, P55 cells cultured under equivalent conditions (route A1); whereby SGR values were (0.021±0.004) and (0.019±0.004). Viability was higher in route B2 over 10 passages with average viability reported as (86.3%±8.1) compared to route A1 (83.3±8.8). The metabolite data demonstrated both culture route B1 (P57 cells seeded at 66,667 cells/cm^2^) and B2 had consistent-specific metabolite rates (SMR) for glucose, but SMR values of route B1 was consistently lower than route B2 (0.00001 mmol, cell-1.d-1 and 0.000025).Results revealed interactions between phenotype, SMR and feeding regime that may not be accurately reflected by growth rate or observed morphology. This implies that current schemes of protocol control do not adequately account for variability, since key cell characteristics, including phenotype and SMR, change regardless of standardized seeding densities. This highlights the need to control culture parameters through defined protocols, for processes that involve culture for therapeutic use, biologics production, and reference lines.

## GRAPHICAL ABSTARCT

**Figure:**
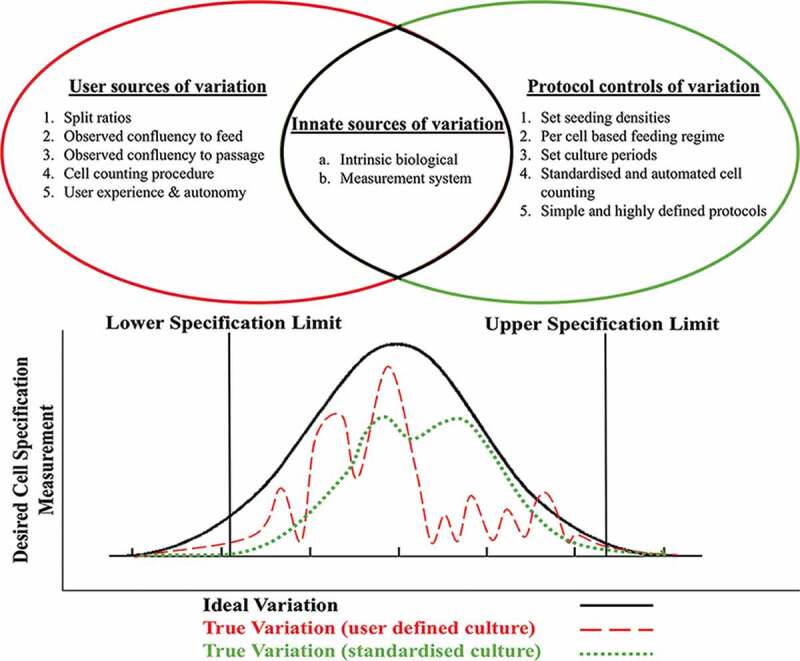

## 1. Introduction

The progression of cell therapies from basic research to clinical products has prompted excitement in the regenerative medicine field; however, challenges still remain to be addressed prior to clinical realization [1–3]. These include the comprehensive characterization of cell therapy products (CTPs), since CTPs are innately complex due to their biological nature. As a result, their characterization is equally complex; unlike traditional pharmaceutical products which have standards that can be easily be produced and used to compare product batches, CTPs currently do not [4–6]. Therefore, reference cell lines are utilized as the closest proxy in CTP manufacturing. However, these reference lines are equally complex and dynamic as the CTPs they are being used to assess. This is exacerbated by the lack of consensus protocol standardization used to culture reference lines, which inherently produces an additional level of uncontrolled variability to the product manufacturing quality control (QC) process [7,8].

Traditional pharmaceuticals assure high purity levels, however, purity presents a challenge for CTPs [5,9], due to the inherent nature of cells which interact with intrinsic and/or extrinsic factors. Unpredictable process variables pose problems where high purity yield is critical to functionality and efficacy. For instance, only ventral midbrain dopaminergic neurons (vmDA) would be required to ensure efficacy and innervation of the correct cell type into the appropriate regions of the dorsal striatum, for the treatment of Parkinson’s disease [10]. Understandably regulators expect proof of high purity, to assure that only cells of interest are procured for patients [11,12]. Achieving high purity with such complex products remains challenging, i.e. ensuring all cells are differentiated or manipulated to the desired state and crucially, retain that state from bench to patient. This is important from a safety perspective and is scrutinized by regulators since undesired cells may potentially cause unwanted/unforeseen effects.

Impurities present another bottleneck which can hinder clinical use and/or commercialization of CTPs. Impurities usually fall into two broad categories: product-related (e.g. cell fragments); and process-related (antibiotics, cell culture reagents *etc*.) [9,13]. Regulators tend to, understandably, have a strict view on all impurities and suggest that they should be addressed in the risk analysis [14]. In addition, impurities including metabolites should be tested and/or have their removal demonstrated through validation. For the most part, the issue of impurities can be addressed by using monoclonal antibodies to remove the cells and leave behind the unwanted fragments using methods such as fluorescence-activated cell sorting (FACS). Furthermore, testing for the absence of bacteria, fungi and mycoplasma is essential [5,9]. In some cases, if accredited, in-house tests can be used to screen the product and this can be integrated into the process quality management of the development and manufacturing process [15]. Importantly, tests and interventions to remove impurities must consider that cells cannot undergo rigorous sterilization and purification steps such as irradiation; that occurs during the manufacturing process of traditional pharmaceuticals. Thus, acceptable limits have been established for (most) CTP process-related impurities; however, the onus is on the manufacturer to set specifications regarding product-related impurities based on factors such: as pre-clinical and clinical safety studies, the capability of the process to remove the impurities and also related products/historical data [14,16,17].

Knowledge of the identity of a product is required, it is crucial that the correct cells are identified and only those that are desired are used for the product. If undesired cells remain in the product and are administered to the patient, they may alter the product’s mechanism of action or result in spontaneous and potentially tumorigenic differentiation, i.e. with remnant pluripotent cells. Cluster of differentiation (CD) markers are one of the most prominent methods of identifying cells, *via* their cell surface molecules [14,18]. However, like many aspects of CTPs there are nuances that present some identification challenges. For instance, similar CD marker combinations can be found on very different cell types; and/or the marker expression profiles can be transient over time. The European Medicines Agency (EMA) says that ‘identity of the cellular components should be based on phenotypic and/or genotypic markers [19]’. As a result, it means that the test methods used need to be specific for the cells of interest. Hence why, when addressing phenotype, relevant analyses should be used such as gene expression, antigen presentation and specific biochemical activity in an orthogonal manner [5,20]. For allogeneic CTPs, it is imperative that the identity profile should include histocompatibility markers, as the cells will be heavily scrutinized by immune system. Tumorigenesis presents trepidation with CTPs, particularly those using heavily manipulated or genetically edited cells, since they may undergo transformation, whereby chromosomal instability is possible [8,19]; thus, impeding authorization. When using unestablished cell lines, karyology tests should be considered to investigate the tumorigenic potential of the cells [19,21,22]. Novel approaches to ensuring CTP safety are being devised, for instance the introduction of a suicide gene can reduce the risk of tumorgenicity for products differentiated from pluripotent cells [23,24].

To this end, the present work highlights the requirement for standardization of culture protocols, especially when the cells are used for QC of cell-based products. This work was executed using the Embryonic Carcinoma (EC) 2102Ep cell line derived from primary human testicular teratocarcinoma [25]. An initial procedure provided by the *National Institute for Biological Standards and Control* (NIBSC) was followed; however, cell growth inconsistencies including population doubling times and growth rates were recorded. Therefore, it is was hypothesized that a major contributing factor to such inconsistency was due to the ambiguity of an undefined protocol that is susceptible to operator interpretation/intervention.

Study-based examples whereby understanding and application of ambiguous protocols that may introduce cell growth inconsistencies into a culture system include operator-specific interpretation of unspecified time points for medium exchange, feeding volume and passage points, that are not based on the number of viable cells/cm [2] in the culture vessels. Commonplace use of split ratio can create inconsistencies regarding when/if the operator intervenes which may ultimately lead to lower yields of the final products and/or process failure. This issue is relevant to many published protocols [7,25–30]. For instance, a range of nonspecific passage timings, i.e. 3–4 days, are suggested for cells to reach ‘adequate’ confluency; observed confluency in itself being highly subjective [31,32]. Additionally, the use of defined seeding densities is absent in many protocols, instead split ratios are prescribed, typically with a range of ratios, i.e. 1:3 or 1:5 [33–36]. Conversely, well-defined feeding and culture protocols in general prevent operator interpretation and therefore would facilitate optimal cell growth and a desired process outcome. The results of a study carried out by Senger *et al.* revealed that optimization of fed-batch parameters, harvest time and feeding strategies of Chinese hamster ovary (CHO) cell cultures *via* well-defined protocols significantly favored cell growth by controlling the metabolite rate in stirred tank reactors [37]. More recent studies have demonstrated that controlling the glycan moieties of antibody therapeutics and improving antibody productivity are highly desirable in maintaining batch-to-batch culture consistency [38]. Kotidis *et al*. employed several strategies to preserve cellular health and productivity while enhancing antibody quality. Their Quality by Design (QbD) approach consisted of a modeling platform and well-defined protocols that allowed for the quantification of the impact of glycosylation precursor feeding on cellular growth, and metabolism in addition to antibody productivity. The platform was then used to optimize feeding strategies that enhanced the final concentration of glycosylated antibody by >90% while sustaining the integrity of viable cell density or final antibody titer [38].

The specific objectives and aim of this work were to identify attributes of a reference cell line that would inform its application including variability in important biological markers under normal laboratory processing variation and key influencing culture parameters.

## 2. Materials and methods

The laboratory setting where these experiments were undertaken run under an industry-style quality system that segregates different cells cultures in to defend, segregated areas and equipment with robust cleaning and maintenance protocols. The facility operates under a quality system based on ISO 9001:2015 quality system principles [39].

### 2.1. In Vitro cell culture

All reagents and consumables were obtained from Fisher Scientific, UK unless otherwise stated. EC 2102Ep (Passage 54, GlobalStem, USA) cells were thawed from cryopreservation and cultured in T25cm^2^ tissue-culture flasks to create a working cell bank. Each vial contained 6 × 10^6^ cells in 1 ml of Cryostor® CS10 (cat# C2874-100ML, Sigma Aldrich, UK) which were stored in liquid nitrogen. The cells were cultured at 37°C, 5% CO2 using Gibco Dulbecco’s Modified Eagle Medium (DMEM) high glucose with GlutaMax™ medium (cat# 10569010) and supplemented with fetal bovine serum (FBS, 10% *v/v*, cat# 10100139) herein referred to as growth medium.

A systematic experimental approach was applied to two parameters: cell seeding density and feeding regime. Initial experiments over three passages were implemented to investigate the effect of these parameters on cell characteristics, with a focus on growth rate, cell viability, phenotype, and specific metabolic rates SMR. These experiments fed into a longer-term 10 passage (30 days) experimental protocols which were utilized to elucidate the long-term effects of the seeding density and feeding regime on the characteristics mentioned above, with the addition of gene expression. The 10-passage duration was implemented in order to compare the overall experimental outcomes to the work of Andrews (1982) that alluded to seeding density being the major driving force for variation in growth and unwanted cell differentiation in EC 2102Ep cells.

### 2.2. Experiment A: four-way comparison of the effects of medium of exchange

**Figure 1.**
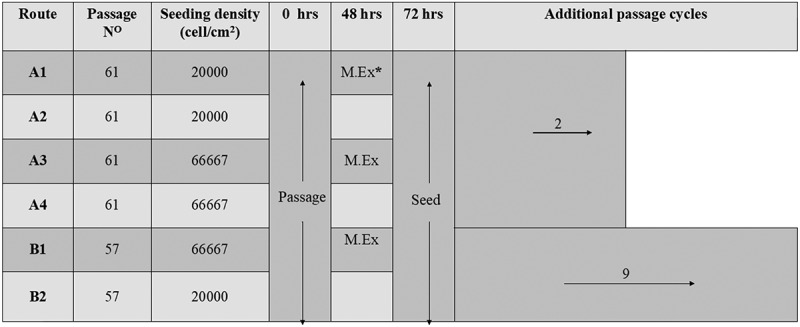
Schematic detailing the experimental culture routes investigated. Cells in each route were seeded according to the density stated in the figure. Following 48 h culture route **A1, A3**, and **B1** were subjected to a 100% medium exchange. Following 72 h, all routes were passaged. Route **A1, A2, A3** and **A4** underwent a further two passages (experiment A). Route **B1** and **B2** underwent a further nine passages (experiment B); *M. Ex = medium exchange

2102Ep cells (P59) were expanded in a T75cm^2^ tissue-culture flask, the growth medium was exchanged following at 48 h. Cells were passaged following 72 h and were seeded into two T75cm^2^ flasks for another expansion passage (P60). The cells were then separated into four different routes of culture (Figure 1): *Route A1* and *A3*, cells were seeded in duplicate at 20,000 and 66,667 cells/cm^2^, respectively in 5 ml growth medium. Following 48 h the growth medium was exchanged. The cells were then passaged following at 72 h; cell counts were obtained in duplicate for each flask and cultured for a further two passages. *Route A2* and *A4*, cells were seeded in duplicate at 20,000 and 66,667 cells/cm^2^, respectively, in 5 ml growth medium. No medium exchange took place prior to passage. The cells were passaged following 72 h culture, cell counts were obtained in duplicate for each flask and cultured for a further two passages.

### 2.3. Experiment B: longitudinal comparison of two protocol culture conditions

2102Ep cells (P55) were cultured in T75cm^2^ tissue-culture flasks; the growth medium was exchanged at48h. The cells were harvested and passaged following 72 h into two T75cm^2^ flasks for another expansion passage (P56). The cells were pooled then separated into two routes of culture in T25cm [2] tissue-culture flasks, herein referred to as B1 and B2 (Figure 1): *Route B1*. Cells were seeded in triplicate at 66,667 cells/cm^2^ in 5 ml of growth medium. Medium was exchanged at 48 h. The cells were harvested and passaged following 72 h (P57). Cell counts were performed in duplicate for each flask and cultured for a further nine passages. *Route B2*: Cells were seeded in triplicate at 20,000 cells/cm^2^ in 5 ml growth medium. No medium exchange took place prior to passage. The cells were then passaged following 72 h culture (P57). Cell counts were obtained in duplicate for each flask and cultured for a further nine passages. Four analytical time points were selected over the 10 passages, two at an early stage of the experiment and two at a later stage (passages 1, 3, 7 and 9).

### 2.4. Cell counting

Cell counts and viability (*via* acridine orange uptake and DAPI exclusion) were obtained using an automated mammalian cell counter (NucleoCounter NC-3000, Chemometec, Denmark). The results were used to obtain the specific growth rate (μ) and the Population Doublings (P_d_) using equations previously published by Heathman *et al.* [40].

### 2.5. Metabolite analysis

Spent media samples, 500 μl, were collected then stored at −20°C prior to analysis. For Experiment A, media samples were collected at two time-points: following medium exchange at 48 h (A1 only) and prior to cell passage. In experiment B, spent media samples were collected on the medium exchange and passage time points for B1 and B3. Samples for route B2 and B4 were collected only on the passage time point. Spent media samples were analyzed for glucose using the Cedex Bio-HT (Roche, Germany). The results were used to obtain the SMR mmol.cell^−1^.d^−1^ (SMR) using an equation previously published by Heathman *et al.* [40].

### 2.6. Flow cytometry

All reagents were obtained from BD Biosciences (Oxford, UK) unless otherwise stated. Flow cytometry immunophenotyping samples were collected and fixed on the day of harvest. Experiment A samples were collected at all three passages. Experiment B samples were collected at passage 1, 3, 7 and 9. A minimum of 1 × 10^6^ cells were collected from culture route and fixed for 20 min in the dark at room temperature (RT, BD Cytofix™). The cells were washed with phosphate buffered saline (PBS) and centrifuged at 500xG (AaccuSpin Micro 17, Fisher Scientific, UK) for 5 min; this step was repeated twice. The cells were permeabilised for 10 min in the dark at RT (a BD Perm/Wash™). Cell staining was performed using pre-conjugated antibodies Oct3/4-PerCp-Cy5.5, SSEA-1-PE and SSEA-4-Alexa 647, for 30 min in the dark at RT, a sample with the respective isotype controls was used to account for nonspecific binding. Following 30 min two washes were performed using the permeabilising buffer. The cells were resuspended in cell stain buffer prior to analysis. In total, 250 μl of each sample was used for analysis using flow cytometry (BD FACSCanto™ II, BD Biosciences, USA).

### 2.7. PCR

PCR samples were collected upon cell harvesting and stored at −80° C until analysis. For experiment B sam ples were collected at passage 1, 3, 7 and 9. Gene expre ssion quantification was performed using Quantitative RT-PCR. Total RNA was isolated using the RNeasy Mini Kit (Qiagen, Cat #74104) following a defined manufacturer’s protocol. RNA yield and purity were determined using the NanoDrop™ 2000 Spectrophoto meter (Thermo Scientific, UK). RNA integrity was assessed using an Agilent 2100 Bioanalyser (Agilent Technologies, Germany). 1 µl of total RNA was reverse transcribed using the QuantiTect Reverse Transcription Kit (Qiagen, Cat #205311) according to the defined manufacturer instructions. 1 µl of the cDNA synthesis reaction was used as template for each real-time PCR using VeriQuest Fast SYBR Green qPCR Master Mix. PCR was run in a StepOnePlus™ Real-Time PCR System (Applied Biosystems, USA) for 40 cycles at 95°C for 3 seconds to denature and 60°C for 30 seconds to anneal. The relative amounts of PCR pro duct were quantified using the relative threshold cycle (ΔΔCt) method corrected for efficiency for each amplification. The gene quantities for each sample were normalized against the geometric mean of expression of the housekeeping genes *GAPDH* and β-actin [25].

#### 2.1. 8. Cell time

Cell time can be applied to quantify the capability of a given volume of medium to sustain the growth of a given number of cells for specific period. Cell time (*CT*) permits the analysis of cell growth medium capacity/medium exhaustion. *CT* is expressed in cell hours/days. Where *N_O_* is the initial cell density, *k* is the specific growth rate and *t* is the time of culture (hours/days). It is the area underneath the curve of a [cell] *vs*. time graph (Equation 1).

(1) $$CT=\left(\frac{N_{o}e^{kt}}{k}-\frac{N_{o}}{k}\right)$$

### 2.8. Statistics

Unless otherwise noted, statistical significance was determined using two-way analysis of variance using Graphpad Prism 7 Version 7.0d (CA, USA). Statistical significance was assigned as indicated in the figure legends. ‘*’ indicates p < 0.05, ‘**’ indicates p < 0.01, ‘***’ indicates p < 0.001, and ‘****’ indicates p < 0.0001. Tukey and Sidak’s multiple comparisons tests were used to compare means between groups. Experiment A; *n*= 3 for each route, *n*= 2 for cell counts, *n*= 2 for metabolite analysis. Experiment B; *n*= 2 for each route, *n*= 2 for cell counts, *n*= 2 for metabolite analysis.

## 3. Results

The aim of this work was to apply parameter changes to an in-house protocol adapted from NIBSC by utilizing defined seeding densities and time-defined passage points. This was to investigate the impact of cell culture parameter changes and highlight the need for standardization; lack of standardization can result in process output variation, i.e. variability of growth rates and cell phenotype.

It was hypothesized that a major contributing factor to such inconsistency was due to the ambiguity of an undefined protocol that is susceptible to operator interpretation/intervention.

The use of defined seeding densities and time-defined passage points were applied to minimize human-based sources of variation including observed confluency and uncontrolled/undefined parameters including split ratios to have a more defined and standardized protocol. The in-house protocol was then applied to a series of ‘quick hit’ experiments performed over three passages to realize the impact of different protocol parameters on characteristic variation including cell metabolic rate, cell-specific growth rates and phenotype. A longitudinal experiment set over 10 passages employed a streamlined version of the in-house protocol, which removed the medium exchange step and used a defined seeding density of 20,000 cells/cm^2^, that differed from the original NIBSC protocol (66,667 cells/cm^2^), to experimentally compare the effect of the changes in protocol.

### 3. 1. Experiment A: four-way comparison of the effects of medium of exchange.

**Figure 2.**
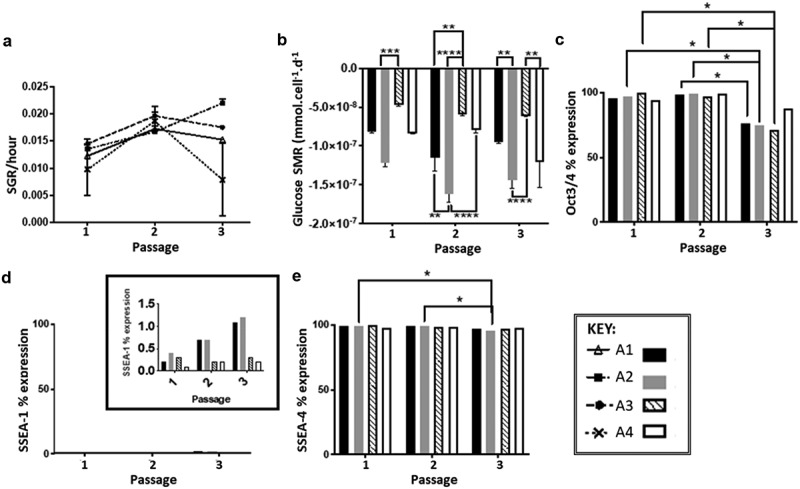
(a) SGR over three passages, similar values are observed in all culture routes, with the exception of A4 at passages 3 (*n*= 2). (b) Glucose SMR over three passages demonstrating that route conditions B2 and B4 that have no medium exchange following 48 h have higher SMR compared to route A1 and A3, significant differences between routes at each passage shown on the graph highlight that seeding density, feeding regime and a combination of both parameters result in a different SMRs (*n* = 2). (c) Oct3/4 marker expression over three passages, expression levels are similar between routes, significant difference observed between passages 2 and 3 for all routes (*p < 0.05) except route A4. (d) SSEA-1 marker expression over three passages, inset shows that percentage expression levels are below 1.5%. (e) SSEA-4 marker expression over three passages, significant differences are observed only within route A2 at the different passages (*p < 0.05). **N.B**. Oct 3/4 and SSEA-4 are positive markers for pluripotent human embryonic stems, SSEA-1 is a negative marker associated with pluripotency of human embryonic stem cells

SSEA-1 and SSEA-4 expression were both stable with minimal variation throughout the three passages, apart from route A2. The cells exhibited variation in Oct3/4 expression in all conditions as expression decreases, most notably at passage three, regardless of whether they were subjected to medium exchange at 48 h; the same trend was observed across all culture routes independent of the seeding density (Figure 2c). Culture route A2 exhibited a higher rate of metabolism (Figure 2b); other significant differences between routes are noted in Table 1. SGR values were similar across all culture routes, independent of culture conditions and density, apart from route A4 at passage number three (Figure 2a).

**Table 1.** Summary of the significance values at each passage, comparing the SMRs of the different culture routes using Tukey’s multiple comparisons test. Both seeding density and feeding regime are shown to result in significant difference between the four routes. The highest significant differences between routes, within passages, are observed when both parameters are changed, e.g. A2 vs A3 (*n* = 2 for each condition)

		Passage 1		Passage 2		Passage 3	
Condition	Parameter	Summary	*P* Value	Summary	*P* Value	Summary	*P* Value
A1 vs. A2	A1 vs. A2	*	0.024	**	0.0082	**	0.0059
A1 vs. A3	A1 vs. A3	*	0.0494	**	0.0026	ns	0.0573
A1 vs. A4	A1 vs. A4	ns	0.998	*	0.0438	ns	0.1793
A2 vs. A3	A2 vs. A3	***	0.0002	****	<0.0001	****	<0.0001
A2 vs. A4	A2 vs. A4	*	0.0327	****	<0.0001	ns	0.2435
A3 vs. A4	A3 vs. A4	*	0.0364	ns	0.3936	**	0.0013

### 3.2. Experiment B: longitudinal comparison of two protocol culture conditions

**Figure 3.**
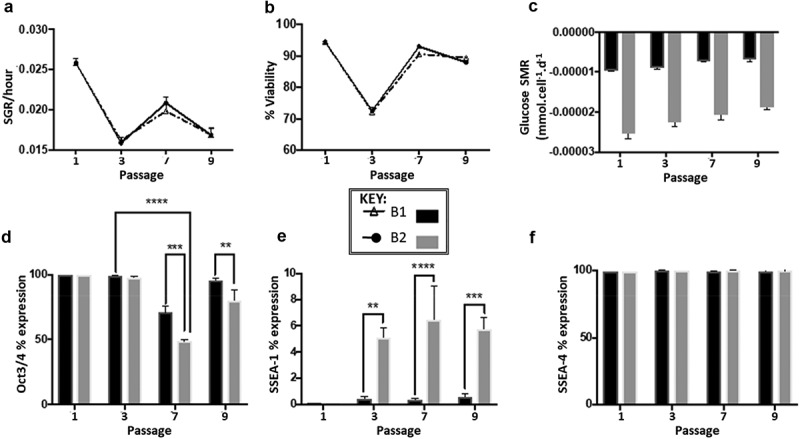
(a) Specific growth rate trend for route B1 and B2 over 10 passages, showing a fluctuation in SGR throughout all 10 passages that follows the same trend regardless of route, *n*= 3 error bars showing standard deviation, SD. (b) Cell viability trend of route B1 and B2 over 10 passages both routes follow a similar trend in viability throughout all passages cycles, route B1 had lower viability during 5 of the passage cycles (4−8) in comparison to route B2, *n*= 2 error bars showing SD. (c) Glucose SMR from passage cycle 2 to 10 demonstrating the significant differences between passages (***p < 0.001), at each individual passage cycle route B2 is significantly higher than route B1 (****p < 0.0001) (d) Oct3/4 marker expression percentages for experiment B at passage cycle 1, 3, 7 and 9 significant differences between passages (****p < 0.0001) and significant differences between culture route B1 and B2 (*p* = 0.0004, *n*= 3). (e) SSEA-1 marker expression percentages for experiment significant differences between culture route B1 and B2 at passages 3,7 and 9, B2 exhibited higher expression levels of SSEA-1 compared to B1. (f) SSEA-4 marker expression percentages for experiment B, no significant differences in expression levels are observed both between passages and within the different routes, B1 and B2

The growth rate data obtained demonstrated that over nine passages, culture route B2 on average had marginally higher SGR values (0.021 ± 0.004) compared to A1 (0.019 ± 0.004), the SGR values of both routes fluctuated throughout the 10 passages (Figure 3a). The fluctuation trend was similar for both routes with regards to SGR values, Pd values and cell viability. Route B2 had a marginally higher average cell viability (86.3% ± 8.1) compared to route A1 (83.3 ± 8.8) over the 10 passages (Figure 3b).

**Figure 4.**
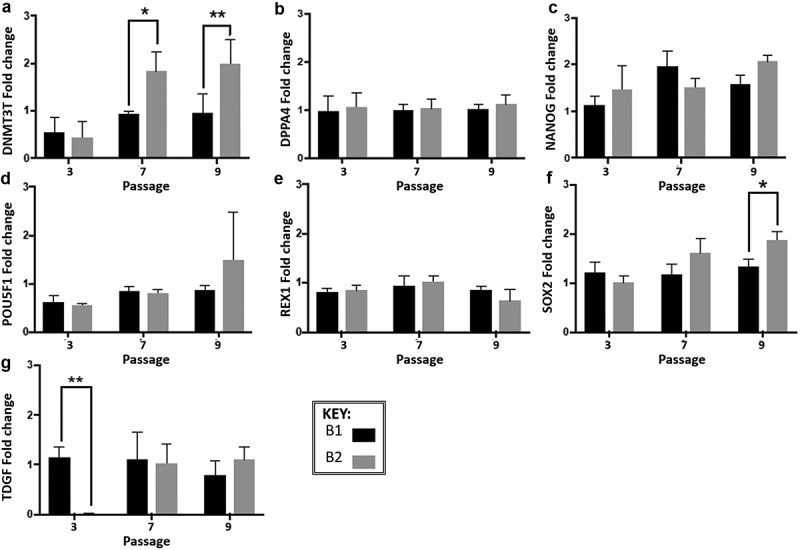
Gene expression analysis of experiment B at three passage points over the 10 passages (normalized to the cells at passage 1), expression was measured by real-time quantitative reverse transcription-polymerase chain reaction (qRT-PCR). Fold change was calculated using the relative quantity of each gene was calculated by the ΔΔCt method, using a correction for the amplification efficiency of that gene, and normalized to the geometric mean of two housekeeping genes: glyceraldehyde-3-phosphate dehydrogenase and β-actin. Significant differences in fold change at different passage cycles were seen only for **A**-DNMT3B (***p = 0.0007), NANOG (*p = 0.01) and SOX2 (**p = 0.004). Significant differences between route A1 and A2 were seen only for **A -**DNMT3B (**p = 0.0032) and **F**-SOX2 (**p = 0.006). Differences between the two routes within a passage cycle were only observed for **G**-TDGF (passage cycle 3, **p = 0.018), **A**-DNMT3B (passage cycle 7, *p = 0.0221; passage cycle 9, **, *p* = 0.0073), and **F**-SOX2 (passage cycle 9, *p = 0.0191). Error bars indicate standard deviation (*n*= 3 for all data presented). **N.B**. Cells from passage 1 were used as the control sample for the qRT-PCR fold change analysis, therefore no data is presented for passage 1 in the graphs

The metabolite data demonstrated that route B1 had a consistent SMR for glucose metabolism over the 10 passages, appearing to be independent of the SGR value (Figure 3a & C). Flow cytometry marker expression analysis showed no significant change in SSEA-4 expression over the 10 passages or between route B1 and B2 (p = 0.07 and p = 0.10, respectively) (figure 3f). However, for route B2 there was expression of SSEA-1 which is a negative marker, this was observed from passage cycle three onwards. Oct3/4 expression in route B2 decreased by 52% and ~30% in B1 at passage cycle seven, which increased back to >95% in route B1 and only to 79.6% by passage cycle nine in route B2 (Figure 3c). The PCR performed for experiment A demonstrated that for a selection of genes analyzed, *DPPA4, POU5F1* and *REX1*, there is no significant difference between the two different routes throughout the 10 passages (Figure 4). However, *DNMT3B, NANOG* and *SOX2* exhibit significant differences between the two routes and between passage cycles as they have higher expression in route B2 (Figure 4a,c,f). *TDGF* is the only gene that had a significant difference at passage cycle three, being much lower than the route B1 condition (** p = 0.018). *DNMT3B, NANOG* and *SOX2* showed differences at passage cycle seven and/or passage nine between the two routes, in all cases route B2 having a higher fold change.

## 4. Discussion

In the context of the growing cell therapy area, it is important to develop robust protocols that are easily transferable and comparable, to ensure high-quality cell products CTPs that satisfy regulatory requirements without being burdensome regarding the manufacturing process [7,8,17]. As such, before truly fully automated and closed systems can be employed, it is important to have standardization in manual and semi-automated protocols that are currently in use. This work set out to demonstrate that defined, specific protocols with specific operating ranges for the variables studied are that are not open to interpretation are necessary to maintain consistency and reduce variability, particularly in the context of reference standards and reference cell lines [27,28,41]. The simultaneous culture of the 2102Ep cells using various culture routes was utilized to explore the effect that changes in protocol parameters have had on the outcome of cell culture process characterization and outcomes.

### 4.1. Effect of variable culture conditions on cell growth variability

Previous work performed in-house has demonstrated that despite using an embryonic carcinoma cell lines like such as 2102Ep, that are considered to be ‘robust’ [42], the growth is inconsistent; however, it is unclear what specifically causes variability in cell culture systems. Environmental and culture factors can have a major effect on growth and cell viability including (sub-optimal) temperature, level of dissolved oxygen, nutrient degradation/depletion, and waste product accumulation [43]. Other prevalent types of inconsistency arise during the measurement and characterization of cell growth. Contributing factors may include a lack of/inadequate calibration of cell counting instruments, poorly defined cell culture and feeding protocols, and a lack of a standardized cell counting instruments in different laboratories [42]. In addition, a lack of QC systems and in-line sensing may result in fundamental and accumulative inconsistences [44] including but not limited to: poor quality of starting materials/stock culture, unsystematic freeze/thaw processing and inadequate mixing of cells, media, and/or other reagents that contribute to poor cell growth and/or cell count inconsistencies [45]. Other pivotal factors to consider are high-passaged cells, incorrect-formulated/poor quality media, buffer and/or serum and microbial contamination (particularly mycoplasma [45]).

The present work hypothesized that the root cause of variability is due to the lack of standardized controlled culture protocols, which results in differences in culture growth and characterization outcomes. It is evident from previous experiments performed by the authors using the 2102Ep cells that, in terms of growth rate the cells are not sensitive to the wide range of densities investigated (5,000 to 93,000 cells/cm^2^) *see Supplementary Data 1 and 2*), illustrating that at least in the case of 2102Ep cells, density is not a limiting factor of cell growth within the relatively wide ranges tested. Instead, it is assumed that the differences observed are a result resultant of the cell system dynamics, as a consequence of protocol parameters such as undefined seeding densities due to split ratio passaging and inconsistencies in feeding regime.

### 4.2. Effect of seeding density and feeding regime on phenotype and SMR

Density has been reported to be a major parameter that can influence undesirable cell differentiation [26,46]. Andrews (1982) previously demonstrated that when 2102Ep cells are seeded at low density (1,300 cell/cm^2^) phenotypical changes arise as evidenced by the expression of SSEA-1 which is a negative pluripotency marker [25,26]. The present work employed a systematic approach to ascertain if density was the predominant factor for differentiation observed by Andrews (1982). The initial experimental design explored feeding regimes as a potential protocol parameter that may attribute to variation, either in isolation or as a combined effect with seeding density. The longitudinal experimental approach investigated the long-term effects of changing both, seeding density and feeding regime parameters. Akin to the work by Andrews (1982), it was observed that changes in seeding density resulted in changes to phenotype and metabolism suggesting cell differentiation; however, it was also observed that the feeding regime employed also resulted in other cell characteristic differences. These differences were assessed using secondary methods of appraisal that are based on SGR and specific consumptions/production of measured metabolites. These analyses revealed that SMR is significantly affected by feeding regime regardless of seeding density, illustrating that controlling seeding in isolation is insufficient for ensuring consistent cell characteristics during cell culture. A limitation of the SMR calculation is that it assumes both a constant cell growth rate and constant consumption/production rate over the measurement period. Any deviation from this will introduce some inaccuracy but will still provide a better estimate than simpler approaches such as dividing change in concentration by average cell density which do not account for the exponential nature of cell growth; thus, it is routinely applied as a standard engineering approach.

Although Andrews (1982) prescribes the route B1 density (66,667 cells/cm^2^) as an optimal seeding density, it is evident that even at this density variation is observed through the passages and experiments carried out. It can be postulated that the variation is due to the innate biological of the cells and the complex cell system dynamics that are involved in culture [4,47,48]. This further illustrates the necessity of protocol standardization to avoid the compounding of different sources of variation.

### 4.3. Stability of cell growth, phenotype, and gene expression in response to streamlined culture protocols

Experiment B was a longitudinal study investigating the stability of the two routes used to assess the effect of feeding regime on cell growth performance. The in-house defined NIBSC protocol condition, route B1, was compared to a streamlined protocol using a lower starting seeding density. The route B2 condition was chosen as previous in-house experiments revealed that cells grown at 20,000 cells/cm^2^ with 5 ml of growth medium performed well in terms of cell viability and SGR (*see Supplementary Data 1 and 2*). The cell time for this condition was then calculated, a value of 2.22 × 10^7^ cell hours per 5 ml of growth medium over 72 h was obtained, which was a more than sufficient medium capacity to sustain the cells over a 72 h culture period without medium exchange. From a manufacturing perspective, this minimal intervention protocol employed is ideal as it reduces operator manipulation and reagent use, which are simple means of lowering operating costs [4,9].

The objective of experiment B was to elucidate whether a cell time-defined protocol, route B2, would permit cells to be cultured in a streamlined manner and retain the same characterization outcomes as the original protocol in terms of growth dynamics, phenotypic expression and metabolism. Josephson *et al.* (2007) previously cultured 2102Ep for several passages and reported no notable characteristic cell changes, which made them an ideal human embryonic stem cell reference line candidate [25]. As such the experiment cultured the cells for 10 passages whilst tracking the growth and metabolic rates along with gene expression and phenotypic markers.

Over the 10 passages of experiment B, a fluctuation trend in growth rate and viability was observed. Interestingly, this fluctuation was synched synchronized between the two different culture routes (Figure 3a,b), suggesting that there is an artifact that synchronizes the two routes in terms of cell growth and viability that is independent of the culture route. It is unclear whether this is an innate feature of the cells (this synchronized behavior is conserved even over 10 passages of the same cell stocks), which alludes to the behavior being an innate cell feature. Alternatively, although not considered to be the sole cause, it could be deemed hypothesized that the synchronized behavior is subject to the result of a measurement system error. This notion is supported by observation of the behavior across independent culture trains. However, different characterization systems (cell counting, immunophenotyping, gene- and metabolic analysis) with distinct manipulation techniques in terms of sample handling were used. Therefore, it is unlikely that measurement error would occur across all techniques, suggesting the synchronized behavior observed is an effect attribute of the cells themselves; especially since the gene expression of *DNMT3B* and *SOX2* also shows the synchronized supports the synched behavior that is seen in the SGR and cell viability (this was illustrated by the no difference in fold change between the two culture routes, and yet there was a significant fold change difference between the passages in the case of DNTM3B, NANOG and SOX2 expression). While there is no difference in gene regulation between the two routes for DPPA4, POU5F1 and REX1 which all had consistently similar levels between routes and between passages (Figure 4b,d,e). The high levels of consistency with the routes throughout the passages showed that when a defined protocol is followed, less variation during culture is observed.

SGR differences were observed for like-for-like conditions between the two experiments, i.e. the A3 condition had a higher SGR at passage three (0.018SGR/h ± 0.004) than at passage 1 (0.015 SGR/h), whilst the opposite was true for the B1 condition. This difference can be attributed to the cells being seeded at different passages. However, this difference is not observed in the SMR, where there cycling trend is similar between the two experiments (Figure 2b,c), suggesting that the observed difference in SGR can be attributed to the innate cell biological variation. Further experimentation would be required to fully ascertain the cause of the observed SGR differences.

The notably higher SMR for route B2 was unexpected as this disparity in SMR has not been previously seen in other published literature or previous work of the authors to the same magnitude. This behavior was observed in experiments A and B, suggesting that changes in SMR are due to the cells being cultured following a streamlined protocol without medium exchange. This implies that SMRs and phenotypic expression are affected by the culture conditions, i.e. nutrient availability based on feeding parameters. The change in expression of SSEA-1 and Oct3/4 further demonstrates that culture conditions have an influence on marker expression. This illustrates the impact of changing the culture environment by manipulating nutrient levels; regardless of previous culture conditions, the cells are the significantly impacted.

The change in expression of SSEA-1 was immediately evident following the first passage, illustrating that feeding regime can impact the cell metabolic behavior and phenotypic expression (Figure 3c,d), but not necessarily the growth performance (Figure 3a). This implies that the potential cause of SMR discrepancy is due to the lack of medium exchange following 48 h, which is substantiated by experiment A, as conditions not subjected to medium exchange had higher SMRs compared to the cells that were subjected to medium exchange. Evidenced through experiment A are the differences between the routes at the three passages, the most significant differences are indicated (Figure 2), other significant differences are reported in Table 1. This highlights that differences in culture protocol based on seeding density, feeding regime, or both, result in notable SMR variation within the same stock of cells cultured simultaneously. The highest significant differences between routes, within passages, are observed when both parameters are changed, e.g. A2 *vs*. A3, where both the seeding density and feed regime are changed.

### 4.4. Cell stability and differentiation in reference cell lines

The EP2102 cells are putatively considered as a reference line, thus it would be assumed that there would be stability in the characterization of the cells. However, over the 10 passages some unexpected behaviors were observed; of particular interest is the presence of SSEA-1 which is a negative marker for pluripotent stem cells, and the decrease in expression of the positive pluripotency marker Oct3/4. The presence of SSEA-1 and the significant decrease in Oct-3/4 (up to 52% in route B2 passage cycle 7) implies that the cells are differentiating (Figure 3c) [25,26,46]. Interestingly, the significant differences in gene expression are observed at passage seven and/or nine, which are the same passage cycles that demonstrate notable differences in phenotypic marker expression through flow cytometry, particularly Oct3/4. It is unclear what causes the change in behavior, in either cell SMR or marker expression [28,49,50].

As previously mentioned, the phenotypic change that has was observed in the experiments could be attributed to cell differentiation. This is a possible effect of the culture conditions, the literature regarding embryonic and pluripotent cells suggests that seeding at low densities causes differentiation, much like the conditions that the cells were cultured under [49–51]. The gene expression negative fold changes observed for DNTM3B and SOX2 suggested that the cells were differentiating as they are key pluripotency markers. Furthermore, the experimental data showed evidence of higher metabolite rate and phenotypic change, which is often concomitant with cell differentiation and/or the presence of a different cell population [52–55]. However, there is evidence within the experiments that is contrary to the cells differentiating, suggesting it is the protocol parameters, i.e. medium exchange that are causing variation as exemplified in experiment A (Figure 2b). For instance, as previously stated, no morphological change was observed under light microscopy throughout the 10 passages.

Another proposed explanation of the observed phenotypic change is that the change is linked to cell death. This rational is reinforced by the fact that the phenotypic change is not cumulative over time, suggesting that the expression is presented just before the cells start to die resulting in non-cumulative expression of SSEA-1. However, cell viability and SGR data show evidence that is contrary to this, as cells in route B2 had on average, higher cell viability and growth rate which would not be expected with cell death (Figure 3a,b). Furthermore, at passage cycle seven there is a drastic decrease in Oct3/4, yet in the next passage there is no significant decrease in cell number or cell viability.

Density had been reported to be a crucial factor in some cell culture protocols, in particular, those using embryonic cell lines, as it is mentioned to have an influence on metabolic behavior and both directed and spontaneous differentiation [51,56–60]. In experiment B the observed differences in behavior could be attributed to the seeding densities used, as the seeding density was one of the main parameters that was altered between the two routes. Nonetheless, density is unlikely to be the only major driving force as previous experiments have shown no drastic difference in SMR and growth based on density (*see online resource 1A, 1 C and 2A)*. Furthermore, the results from experiment A demonstrated that growth medium exchange, the second parameter, has a significant effect on SMR regardless of density. This implies that cell metabolism based on feeding regime has a notable impact on SMR and marker expression I addition to seeding density. It is well understood and recognized that metabolic rates are a function of nutrient availability [61,62]; it is therefore unsurprising that conditions with a higher cell load exhibit a lower metabolic rate measured by SMR as nutrient depletion will result in decreased nutrient availability overall. Thus, if these parameters are not adequately controlled the resultant cell output quality would be highly variable and incomparable between different process runs and undoubtedly between different sites, which would compromise the use of the cells be it as a reference standard, or therapeutic product. This highlights the necessity of standardized culture procedures to maintain consistency and reduce variability.

### 4.5. The requirement for standardized culture protocols for maintenance of cell quality

The above rationalizations highlight that even in a cell line that is considered as a stable reference point [25,26,30], differences can be observed due to changes in parameters and conditions, over time. These different parameters, such as when to perform medium exchanges and the use of seeding densities that vary, i.e. through the use of non-standardized seeding densities or split ratios, are often left to the operator’s discretion in many protocols. Evidently, this results in noteworthy effects on cell behavior and characterization outcomes. Split ratios are not best practice as innately cells will grow differently from passage to passage. For example, a split ratio of 1:3 can be drastically different from passage to passage, particularly if the cells grow at significantly different rates. In addition, split ratios that are based on observed confluency, are also likely to cause variation in cell growth dynamics. This is due to the subjective nature of observed confluency and its lack of accurate representation of cell number, therefore potentially resulting in non-ideal metabolite profiles that can influence cell growth behavior, producing further cell system inconsistency. Differing levels of variability and non-conformity are detrimental to the successful use of reference cell lines, especially for those intended to be a standardized QC reference.

During the process development and training, operators should record possible areas for error, process deviation and associated risks using a centralized database, overseen by a qualified person (QP) [42]. All operators are assumed to have successfully completed standardized training and assessment of cell culture practice, liquid handling, aseptic technique and equipment operation. Also, they must be able to demonstrate competence in the assessment of any process against quality attribute (QA) criteria using appropriate assays and techniques [63]. Protocols should be written to minimize operator’s discretion and limit the content to the technical ability to run the assays and equipment. Furthermore, detailed standard operating protocols (SOP) inclusive of risk mitigation strategies should be referred to for all procedures and equipment (including maintenance/calibration) [42]. This is a requirement of all laboratory users within not only our facility but all institutions working to Good Laboratory Practice (GLP) guidelines [27,64,65].

This is of relevant importance as the use of reference cell line has seen prominence due to the increase in chimeric antigen receptor T-cell (CAR-T) usage [66,67], which use them as QC reference standards for flow cytometry [4]. Interestingly, flow cytometry analysis revealed marker profile differences; even when the cells were at the prescribed density of 66,667 cells/cm [2], showing that despite a consistent culture process and protocol there is inherent variation in marker profiles through the 10 passages.

This further emphasizes the need to standardize cell seeding within protocols to minimize and control variation as much as possible. This can be achieved by obtaining cell counts, ensuring that the input and output cells numbers are used to maintain optimal culture conditions for growth, without entering regions of metabolic instability. Consequently, this allows for greater control and consistency of the culture system as the seeding density and nutrient levels are predefined. The use of cell time, which is a concept that can be used to quantify the capability of a given volume of medium to sustain the growth of a given number of cells for a specific period, ensures that the culture system given the set density and nutrient availability will not enter a region of metabolic strain. This is important since imbalances in key nutrients including glucose levels have previously been shown to result in limitation of cell growth [7,60], and importantly for reference lines, impact the stability of marker expression. Metabolic strain is potentially problematic if not controlled, as reference lines are used for QC of CTPs need to demonstrate their stability over time, using gene and immunocytochemistry marker expression.

It has been illustrated that cell system behavior is affected by protocol parameters; experiments A and B demonstrated that feeding regimes (medium exchange) had a significant effect on SMR and phenotypic marker expression when compared to seeding density. This highlights that differences in culture parameters ***do*** cause variation; however, it was evident that when parameters were controlled that less variation occurred. Prior experiments detailed in the supplementary information (*Online resource 1*) showed that there was no difference in SGR between different seeding densities when the feeding regime was maintained throughout the culture period. It has been demonstrated through a range of experiments that density was not the sole factor that can cause variation especially when the flasks were seeded at the same density. Instead it was clear that other protocol parameters themselves or when compounded with seeding density resulted in variation. Here it has been demonstrated that both density and the feeding regime had a major effect on cell culture variation. Lower densities have been shown to have distinctly different behaviors compared to higher-density cultures in terms of their SMR, a combinatory combined effect of density and feeding regime has been shown to cause variation in SGR, SMR, phenotype and expression of some genes. However, it is unclear under what mechanism medium exchange or lack of medium exchange influences cell characteristics such as SMR and phenotypic marker expression.

This work provided a demonstration of biological variation arising when culturing a reference cell line with pluripotent attributes under normal laboratory operating controls. It also identified the extent to which key culture parameters contributed to this variation and therefore are critical to control for appropriate application of a reference cell line. Systematic culture trends and patterns observed in biological markers were identified that, if not accounted for, would lead to noise in ruler line applications. These attributes were identified for a range of key markers including cell number, metabolic behavior, surface markers and gene expression.

## 5. Conclusion

The present work illustrates how culture conditions impact cell characteristics, notably, SMR and phenotype. This demonstrates the complex interactions between gene expression, phenotype and feeding regimes that cannot be accurately represented by growth rate and cell counts alone. The lack of robust, well-defined, and standardized protocols results in compounded variation in culture systems. This is due to differences in inter-lab/individual decision-making processes based upon protocol parameters, including observed confluency to gauge when to perform a cell passage. This highlights that non-standardized, ambiguous protocols can easily generate differences over time. At present, this work is yet to be repeated and the results confirmed in other laboratory environments. However, it will provide the basis for others to do so in the near future since standardized protocols are crucial not only from a uniformity perspective but also for ensuring process reproducibility and comparability; particularly in decentralized manufacturing of cell-based products. This is integral to the pragmatic and successful use of reference lines in cell therapy manufacturing and the manufacturing of CTPs in general.

The present work demonstrates that culture conditions have a significant impact on cell characteristics, notably on specific metabolic rate SMR and phenotypic marker expression. Thus, demonstrating that there is a. This demonstrates the complex interactions between gene expression, cell phenotype and the feeding regimes that cannot be accurately represented by growth rate and cell counts alone. The lack of robust, well-defined and standardized cell-culture protocols result in compounded variation in the culture systems. This is due to differences in inter-lab and individual decision-making processes based upon protocol parameters, such as including observed cell confluency as a to gauge of when to perform a cell passage. This highlights that use of non-standardized, ambiguous protocols can easily produce differences over time, illustrating the need for standardization. standardized protocols. Such standardized Standardized protocols are crucial not only from a uniformity point of view perspective but also for ensuring the reproducibility and comparability of a process particularly in decentralized manufacturing of cell-based products. The point of reproducibility is integral to the pragmatic and successful use of reference cell lines in cell therapy manufacturing and the manufacturing of CTPs in general.

## Supplementary Material

Supplemental Material

## Research Highlights

- Subtle, often unintentional variations in culture parameters have a significant impact on cultured cells.
- The importance of balancing key nutrients, gene expression, phenotype and feeding regimes was shown.
- Standardization is crucial to ensure manufacturing process reproducibility and comparability.
- This is integral to the pragmatic and successful use of reference lines in cell therapy manufacturing.
- Our experimental methodical approach demonstrates the complex interactions and balance required between key nutrients, gene expression, cell phenotype and feeding regimes employed that cannot be accurately represented by growth rate and cell counts alone.
- Standardised protocols are crucial not only from a uniformity perspective, but also for to ensure process reproducibility and comparability, particularly in the decentralised manufacturing of cell-based products.
- This is integral to the pragmatic and successful use of reference cell lines in cell therapy manufacturing.
