# Supplementary material for: The importance of cell culture parameter standardization: an assessment of the robustness of the 2102Ep reference cell line
Source: Bioengineered. 2021 Jan 11;12(1):341–57. doi: 10.1080/21655979.2020.1870074 (PMC8806261; doi:10.1080/21655979.2020.1870074)
Supplement: Supplemental Material [file KBIE_A_1870074_SM4427.zip › supplement/Supplementary Material_Bioengineered_revisedclean.docx]

Supplementary Material

**Supplementary Information**

The supplementary information demonstrates how a range of prior experiments were performed to elucidate which protocol parameters had an effect on characteristic variation during 2102Ep cell culture.

Experiment 1: Growth analysis of four seeding densities

The experiment was designed to assess how the cells would grow under a range of different seeding densities ranging from 5,000-20,000 cells/cm^2^. The cells exhibited a typical growth curve over the 96 h culture period. With the exception of cells seeded at 10,000 cells/cm^2^ at day 1, the specific growth rate (SGR) was similar between the different conditions. Cells seeded at 10,000 cells/cm^2^ were observed to have a much higher SGR on day 1(0.029 h^-1^) which dropped on day 2 to align with that other conditions (0.016 h^-1^). At day 4 the observed growth curve illustrates that all the conditions were still in a phase of growth or just beginning to plateau, with 10, 15 and 20,000 cells/cm^2^ all having similar rates from day 2 to day 4 (**Fig. 1A**). For all 4 densities there were no detrimental effects on the viability of the cells. With the exception of cells seeded at 5,000 cells/cm^2^, all conditions had higher percentage viability at day 4 compared to day 2 and 3 (**Fig. 1B**). Metabolite analysis shows a relationship between SGR and specific metabolite rate (SMR) i.e. the cells consumed more glucose as the cell number increased (**Fig. 1C**)


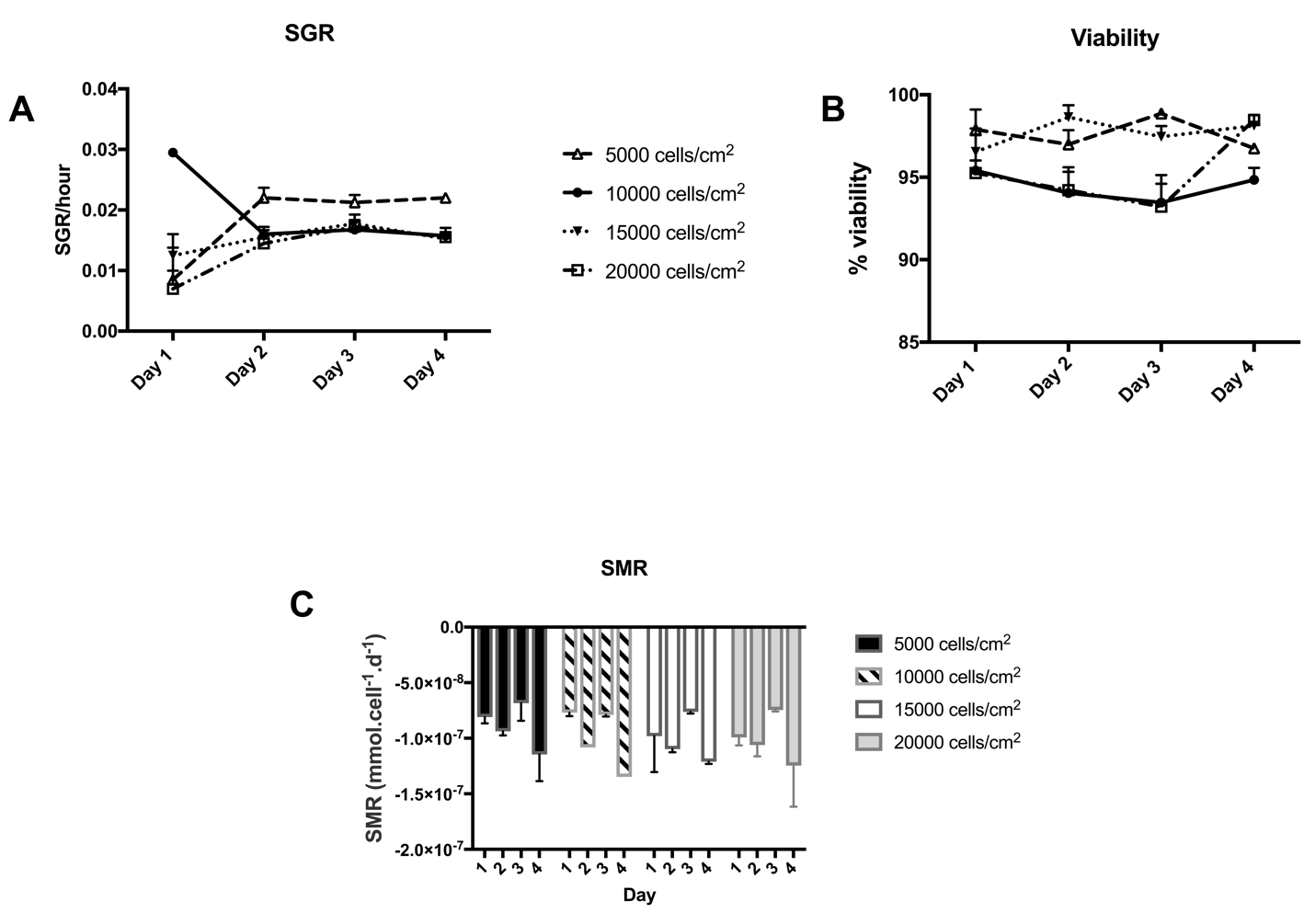


**Fig. 1**: (**A**) Specific growth (SGR) trend for the 4 different seeding densities, e, n=4 error bars showing standard deviation, SD. (**B**) Cell viability trend over 96 hour culture period, all conditions are above 90 % viable throughout the culture period, n=4 error bars showing SD. (**C**) Glucose SMR for all 4 seeding densities, no significance difference observed throughout the culture period or between different densities, n=2 error bars showing SD.

*.*

*Experiment 2: Growth analysis of 6 densities*

Experiment 2 explored a wider range of densities as denoted by the legend, 66,667 cells/cm^2^ was used as this is the density that is recommended by NIBSC; 80,000 cells/cm^2^ was chosen in order to investigate how far the cells could be stressed by seeding density. The other densities were kept the same in order to understand if the same trends from experiment 1 would be observed. The three higher densities (40,000, 66,667 and 80,000 cells/cm^2^) had a decline in cell number resulting in a neutral/ negative growth rate (-0.019 to 0.006 h^-1^), see **Fig. 2A**. This is not likely to be due to cell death as the cell viabilities were all above 90% on day 1 (**Fig. 2B),** this observation is most likely due to a seeding error at the beginning of the experiment, resulting a ~ 30% decrease in cell number from 0 to 24 h. The three higher density conditions behaved similarly to each other, whilst the 3 lower densities flasks also behaved similarly to each other, suggesting a grouping of cell growth behaviour dependent of seeding density. The SGR for the lower densities (5,000 and 10,000 cells/cm2) were on average slightly lower (0.021 h-1) over the 3-day period, whilst the higher densities had an average μ of 0.022 h-1. Overall the cells grew exponentially and did not hit a phase of decline or inhibition within the 7 h culture period, independent of cell density as the SGR between conditions was not significantly different at day 2 and 3. The metabolite data revealed that the main significant differences are between the 5,000 cell/cm^2^ condition and all the other conditions at day 1. At day 2 and 3 notable differences in SMR were only observed between the 3 low densities and 3 higher densities (**Fig. 2C)**, again highlighting a grouped behaviour pattern as seen in the SGRs.


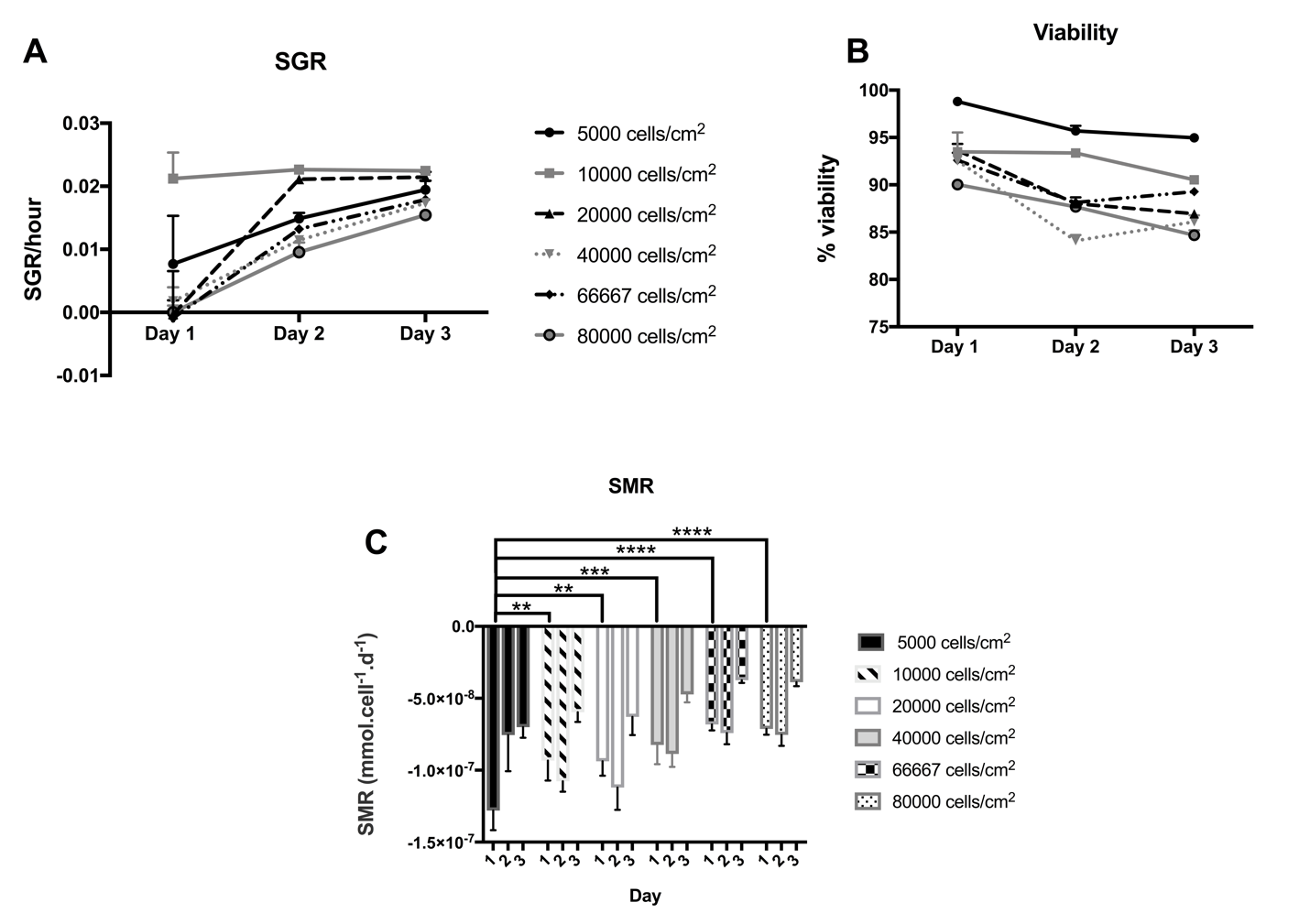


**Fig. 2** Data was collected every 24 h over a 72h culture period, 6 difference seeding densities were investigated in the experiment. Error bars indicate standard deviation, n=3 for each condition; n=3 for SMR data; n=9 for SGR and cell viability data. (**A**) SGR is shown to increase for all 6 conditions over the culture period. (**B**) Cell viability is decreases uniformly for all conditions from day 1 to day 3. (**C**) Significant differences between conditions are at day 1, between 5,000 cells/cm^2^ and the other densities. “**” indicates p< 0.01, “***” indicates p< 0.001, and “****” indicates p<0.0001

Table 1: Experiment 2 SGR. Tukey’s multiple comparisons test of the mean SGR in each condition compared the means of the other conditions on that day. Summary shows only the conditions with significant difference, at day 1 it is evident that the 10,000 cells/cm^2^ condition has a higher SGR than the other conditions. At day 2 only 2 of the conditions are difference and no differences between conditions are noted at day 3.

| **Experiment 2 SGR** | | |
| --- | --- | --- |
| **Day 1** | **Summary** | **P Value** |
| 5,000 cells/cm^2^ *vs.* 10,000 cells/cm^2^ | * | 0.0119 |
| 10,000 cells/cm^2^ *vs.* 20,000 cells/cm^2^ | **** | <0.0001 |
| 10,000 cells/cm^2^ *vs.* 40,000 cells/cm^2^ | **** | <0.0001 |
| 10,000 cells/cm^2^ *vs.* 66,667 cells/cm^2^ | **** | <0.0001 |
| 10,000 cells/cm^2^ *vs.* 80,000 cells/cm^2^ | **** | <0.0001 |
|  |  |  |
| **Day 2** | **Summary** | **P Value** |
| 10,000 cells/cm^2^ *vs.* 80,000 cells/cm^2^ | * | 0.0168 |

Table 2: Experiment 2 SMR. Tukey’s multiple comparisons test of the mean SMR in each condition compared the means of the other conditions on that day. Summary shows only the conditions with significant difference, at day 1 it is evident that the 5,000 cells/cm^2^ condition has a higher SMR than the other conditions. At day 2 the differences observed in SMR are mainly between the lower densities and the higher densities, elucidating to the group behaviour shown in Figure 2A and 2C. The lowest density and the two highest densities are the only conditions noted to be different at day 3.

| **Experiment 2 SMR** | | |
| --- | --- | --- |
| **Day 1** | **Summary** | **P Value** |
| 5,000 cells/cm^2^ *vs.* 10,000 cells/cm^2^ | ** | 0.0046 |
| 5,000 cells/cm^2^ *vs.* 20,000 cells/cm^2^ | ** | 0.0054 |
| 5,000 cells/cm^2^ *vs.* 40,000 cells/cm^2^ | *** | 0.0001 |
| 5,000 cells/cm^2^ *vs.* 66,667 cells/cm^2^ | **** | <0.0001 |
| 5,000 cells/cm^2^ *vs.* 80,000 cells/cm^2^ | **** | <0.0001 |
| **Day 2** | **Summary** | **P Value** |
| 5,000 cells/cm^2^ *vs.* 10,000 cells/cm^2^ | * | 0.0105 |
| 5,000 cells/cm^2^ *vs.* 20,000 cells/cm^2^ | ** | 0.0027 |
| 10,000 cells/cm^2^ *vs.* 66,667 cells/cm^2^ | ** | 0.0067 |
| 10,000 cells/cm^2^ *vs.* 80,000 cells/cm^2^ | ** | 0.01 |
| 20,000 cells/cm^2^ *vs.* 66,667 cells/cm^2^ | ** | 0.0017 |
| 20,000 cells/cm^2^ *vs.* 80,000 cells/cm^2^ | ** | 0.0025 |
| **Day 3** | **Summary** | **P Value** |
| 5,000 cells/cm^2^ *vs.* 66,667 cells/cm^2^ | ** | 0.0089 |
| 5,000 cells/cm^2^ *vs.* 80,000 cells/cm^2^ | * | 0.0136 |

The SGR values observed in the present work were comparable with previous literature published using Human pluripotent embryonic stem cells (hESC), specifically the H9 cell line ^1^ and the hESC cell lines MEL1 and MEL 2 ^2^ (**Table 3**). This demonstrates the association between glucose SMR and metabolism dependent upon the culture conditions and protocols followed.

**Table 3**: Evaluation of Glucose SMR of cells cultured under Route B1 and B2 in comparison to previously published studies using similar cell types

| **Cell type** | **Glucose SMR**  **(mmol.million cell.min)** | **Study** |
| --- | --- | --- |
| Embryonic Carcinoma (EC) 2102Ep cell line derived from primary human testicular teratocarcinoma  EP2102 (route B1)  EP2102 (route B2) | 0.00001  0.000025 | Present work |
| Human pluripotent embryonic stem cells (hESC)  H1  H9 | 0.000071  0.000028 | Gu et al 2016 ^1^ |
| hESC MEL1 and MEL2 (Australian Stem Cell Centre, Monash, Vic., Australia)  MEL1  MEL 2 | 0.000066  0.000083 | Lees et al 2015 ^2^ |

1. Gu W, Gaeta X, Sahakyan A, Chan AB, Hong CS, Kim R, Braas D, Plath K, Lowry WE, Christofk HR. Glycolytic Metabolism Plays a Functional Role in Regulating Human Pluripotent Stem Cell State. Cell Stem Cell 2016; 19:476–90.

2. Lees JG, Rathjen J, Sheedy JR, Gardner DK, Harvey AJ. Distinct profiles of human embryonic stem cell metabolism and mitochondria identified by oxygen. Reproduction [Internet] 2015 [cited 2020 Dec 17]; 150:367–82. Available from: https://pubmed.ncbi.nlm.nih.gov/26159831/

**Table 4:** Instrumentation utilized for experimental protocols and analysis of the effect of different culture parameters

| **INSTRUMENT** | **SENSITIVITY, ACCURACY & DETECTION RANGES** | **QUALITY ASSURANCE PROCEDURES** |
| --- | --- | --- |
| Cedex Bio HT Analyzer (Roche, Germany) | Min detection limit for glucose: ≥ 36.03 mmol/L  Reaction volume 120 - 245 μL  Wavelength range: 340 - 800 nm, 12 wavelengths  Mono- and biochromatic measurement  Measuring range 0 - 2.0 A (for 5 mm path length)  Measuring range: glucose: 36.03- 900.90 mmol/L | All operators to have completed standardized in-house training and demonstrated competence to an onsite qualified person (QP).  Operator to follow detailed SOP when performing assay/analysis  Instrument sensitivity and accuracy is dependent on each metabolite kit, but recovery is: ±10% |
| StepOnePlus™: Real-Time PCR System (Applied Biosystems, USA) | Capacity: 96 x 0.1 ml tubes, 12 x 8-tube strips, 1 x 96-well plate  Optics: 4 emission filters, Blue LED excitation source, photodiode  Peak Block Ramp Rate: 4.6°C⁄sec  Reaction Volume Range: 10-40 µl  Run Time: <2 hrs⁄run (Standard Mode), <40 min⁄run (Fast Mode)  Sample Ramp Rate:Fast mode: ± 2.2°C⁄sec, Standard mode: ± 1.6°C⁄sec  Temperature Accuracy: 0.25°C (35 to 95°C) of display temperature  Temperature Range (Metric): 4-100°C  Temperature Uniformity:0.25°C (35 to 95°C) of setpoint/display temperature  Dynamic Range: Linear Dynamic Range greater than 9 log units (detection)  Sensitivity: 1 copy | All operators to have completed standardized in-house training and demonstrated competence to an onsite QP.  Operator to follow detailed SOP when performing assay/analysis  All SOPs written/followed according to the StepOne and StepOnePlus Maintenance and Administration user guides  StepOne and StepOnePlus Maintenance and Administration Guide Dye: VIC®, SYBR® Green I, ROX™, JOE™, NED, FAM™, TAMRA™  Each StepOnePlus™ Real-Time PCR System is factory-calibrated for optical and thermal accuracy  Calibration plates can be stored and reused three times for up to one year provided they are returned to original packaging and stored at -20°C |
| NanoDrop™ 2000 Spectrophotometer (Thermo Scientific, UK) | Wavelength Accuracy: +1 nm  Spectral Resolution: <1.8 nm (FWHM @Hg 253.7 nm)  Absorbance Precision: 0.002 absorbance (1 mm path)  Absorbance Accuracy: ± 2% (at 0.76 absorbance at 257 nm)  Absorbance Range: 0.02 -300 (10 mm equivalent)  Detection limit: 2 ng/µL dsDNA  Maximum Concentration: 15,000 ng/µL (dsDNA)  Measurement Time: < 5 seconds | All operators to have completed standardized in-house training and demonstrated competence to an onsite QP  Operator to follow detailed SOP when performing assay/analysis    The instrument’s auto-ranging pathlength reduces the need for sample dilution, thus reducing operator adjustment during preparation of samples |
| BD FACSCanto™ II, BD Biosciences, USA)  *8-Color option with 3 Lasers* Fluorescence Detectors 8 PMTs in 4-2-2 configuration Laser Dyes: Violet: Pacific Blue™, AmCyan (455, 488 nm) Blue: FITC, PE, PerCP or PerCP-Cy5.5, PE-Cy7 (525, 575, 678 or 695, 785 nm) Red: APC, APC-Cy7 (660, 785 nm)*  *Detector Bands: Violet: 450/50; 502 to 525 nm Blue: 530/30; 585/42; >670; 780/60 nm Red: 660/20; 780/60 nm* | Carryover: ≤0.1%  Sample Injection: direct into flow cell  Max Particle Size: 50 μm  Sample Flow Rate, Min: 10 μL/min  Sample Flow Rate, Max: 120 μL/min  Sample Acquisition Rate: 10,000 events/second, 6 compensated fluorescence parameters and 2 scatter parameters  Fluorescence Threshold Sensitivities: FITC <100 MESF; PE <50 MESF  Sample Dead Volume: 30 μL (BD Falcon™ tubes 12 x 75-mm) | All operators to have completed standardized in-house training and demonstrated competence to an onsite QP  Operator to follow detailed SOP when performing assay/analysis  Fixed alignment flow cell minimizes start-up time and improves reproducibility  Optical design maximizes signal detection and increases sensitivity and resolution for each colour in a multicolour assay  Automated daily routine procedures including start-up/shutdown, and cleaning cycles  BD FACS™ shutdown solution prevents salt crystal buildup in fluidics lines and is supplemented with a preservative to prevent bacterial growth  The shutdown solution replaces sheath fluid in all sample and sheath fluid lines  LED signal to alert operator when levels reac a prescribed threshold |
| NucleoCounter NC-3000 (Chemometec, Denmark) | Optimal Range: 5 x 10^4^ – 5 x 10^6^ cells/ml (for counting)  Analysis volume: 0.8-16µl and 20-40 µl  Cell types: Mammalian cells, yeast, insect cells, avian cells  Optics Lens with x 2 magnification, 1/2” CCD with 1392 x 1040 pixels  8 integrated light sources (7 of them illuminating from 365 nm to  625 nm and a white light source) and with 9 emission filters | All operators to have completed standardized in-house training and demonstrated competence to an onsite QP  Operator to follow detailed SOP when performing assay/analysis  After transportation the Installation Qualification (IQ) protocol is initiated. The protocol inspects critical internal alignment and report any changes. The Operation Qualification (OQ) protocol verifies correct operation of the instrument |
