# Supplementary figures and images for: The importance of cell culture parameter standardization: an assessment of the robustness of the 2102Ep reference cell line
Source: Bioengineered. 2021 Jan 11;12(1):341–57. doi: 10.1080/21655979.2020.1870074 (PMC8806261; doi:10.1080/21655979.2020.1870074)

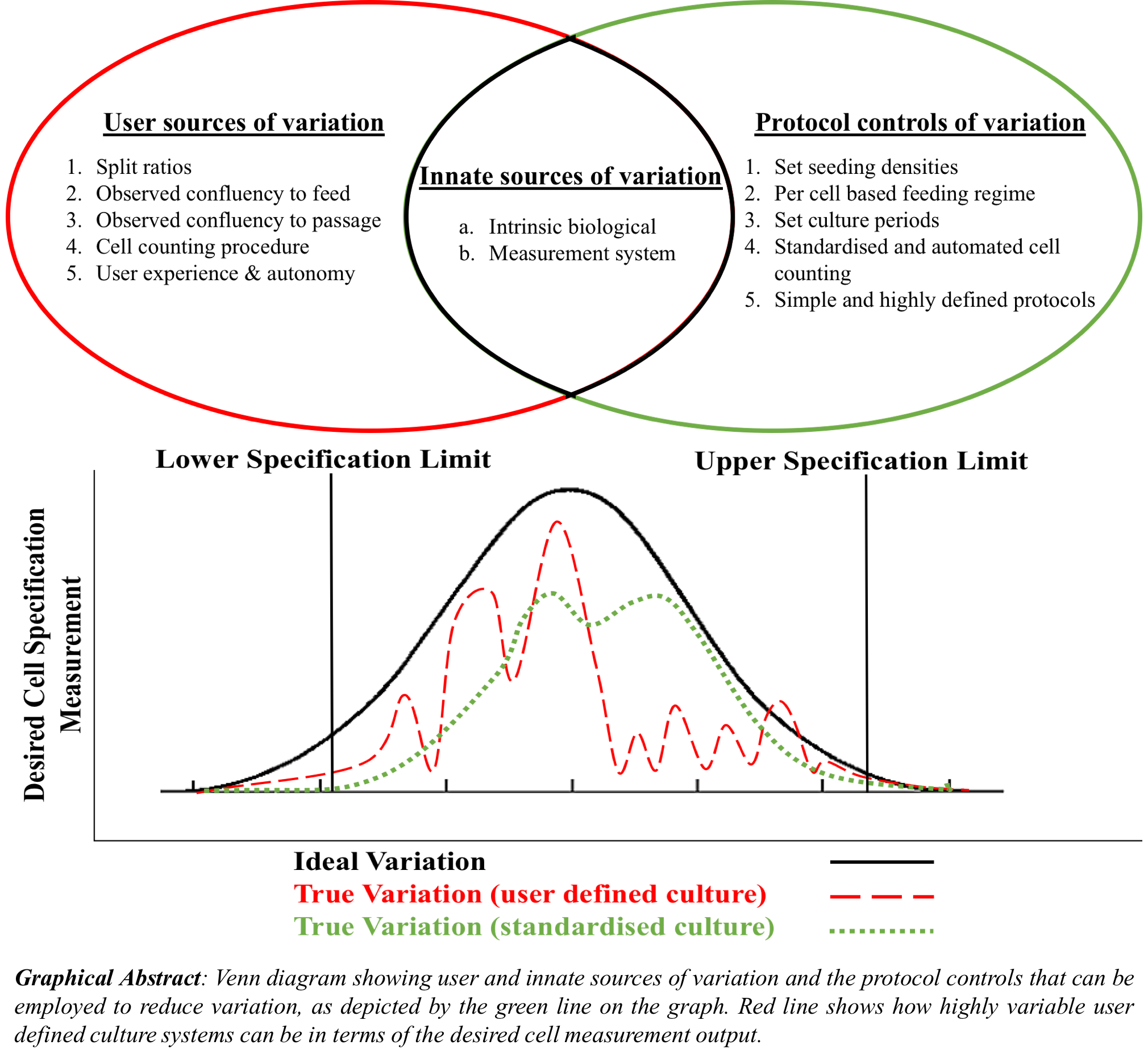

Supplement: Supplemental Material [file KBIE_A_1870074_SM4427.zip › supplement/Graphical Abstract JK.tiff]
